# Supplementary material for: Mapping the evolution of fertility support policies in China: A content and instrumental analysis
Source: PLoS One. 2025 Oct 9;20(10):e0332137. doi: 10.1371/journal.pone.0332137 (PMC12510515; doi:10.1371/journal.pone.0332137)
Supplement: S1 Appendix — (ZIP) [file pone.0332137.s001.zip › S1 Appendix. 226 original policy documents/81-国务院关于印发国家人口发展“十二五”规划的通知(FBM-CLI-2-165987).doc]

国务院关于印发国家人口发展“十二五”规划的通知

**发布部门：** [国务院](https://www.pkulaw.com/law/chl?Aggs.IssueDepartment=5)

**发文字号：**国发〔2011〕39号

**发布日期：**2011.11.23

**实施日期：**2011.11.23

**时效性：** [现行有效](https://www.pkulaw.com/law/chl?Aggs.TimelinessDic=01)

**效力级别：** [国务院规范性文件](https://www.pkulaw.com/law/chl?Aggs.EffectivenessDic=XC0203)

**法规类别：** [人口计划管理](https://www.pkulaw.com/law/chl?Aggs.Category=09701)

国务院关于印发国家人口发展“十二五”规划的通知
（国发〔2011〕39号）

各省、自治区、直辖市人民政府，国务院各部委、各直属机构：
　　现将《国家人口发展“十二五”规划》印发给你们，请认真贯彻执行。

国务院
二○一一年十一月二十三日

　　国家人口发展“十二五”规划

　　本规划根据《[中华人民共和国国民经济和社会发展第十二个五年规划纲要](https://www.pkulaw.com/chl/603c7a7f2158b52bbdfb.html)》和《中共中央[国务院关于全面加强人口和计划生育工作统筹解决人口问题的决定](https://www.pkulaw.com/chl/5e7b01010a4102ffbdfb.html)》（中发〔2006〕22号）精神制定，旨在阐明“十二五”时期国家人口发展的基本思路、发展目标和工作重点，是指导未来五年我国人口发展的纲领性文件，是各级政府和部门全面做好人口工作的重要依据。
　　**一、**“十二五”时期人口发展形势
　　（一）“十一五”时期人口发展的成就。
　　生育水平继续保持稳定。人口计生工作思路方法不断创新，更加注重利益导向，更加注重服务关怀，更加注重宣传倡导，稳定低生育水平长效机制基本建立。以计划生育家庭奖励扶助制度、“少生快富”工程和特别扶助制度为主体的利益导向政策体系初步形成。计划生育优质服务和生育关怀行动普遍开展。婚育新风进万家活动、关爱女孩行动和新农村新家庭计划深入推进。妇女总和生育率稳定在1.8以下，人口自然增长率保持在6‰以内，2010年末总人口为13.41亿人。
　　人口素质稳步提高。科技、教育、人力资源、文化、卫生、人口计生事业不断发展。各级教育普及水平进一步提高，城乡免费九年义务教育全面实现，2010年高等教育毛入学率达到26.5％，15岁以上国民平均受教育年限达到9年，新增劳动力平均受教育年限达到12.4年。人才队伍建设成效显著，人才资源总量不断扩大。孕产妇和婴儿死亡率继续下降，人均预期寿命达到73.5岁。
　　人口城镇化水平继续提高。积极稳妥推进城镇化，促进农业转移人口转为城镇居民。2010年城镇人口6.7亿人，城镇化率达到50％。流动人口服务管理体制建设取得积极进展。
　　城乡就业规模持续扩大。积极就业政策取得成效，城乡就业形势总体稳定。城镇新增就业5771万人，转移农业劳动力4500万人，城镇登记失业率保持在4.3％以下。
　　民生建设取得新进展。城乡居民收入快速增长，生产生活条件得到改善。城镇居民基本医疗保险制度全面建立，新型农村合作医疗制度全面实施；新型农村社会养老保险试点启动并逐步扩大范围，全国统一的城镇企业职工基本养老保险关系转移接续办法出台，企业退休人员基本养老金不断提高；生育保险制度进一步完善。城乡居民最低生活保障全面加强，重点优抚对象抚恤优待标准大幅度提高。妇女和未成年人权益保护得到加强，残疾人参与社会生产生活的环境进一步改善，老年人社会保障和服务水平不断提高。减灾救灾和应急管理体制机制进一步健全。
　　（二）“十二五”时期人口发展的阶段性特征。
　　人口增长势头减弱。生育旺盛期妇女数量开始呈现下降态势，同时随着人口老龄化程度提高，人口死亡率有所上升，在生育政策不变的条件下，人口增长的势头进一步减弱。
　　主要劳动年龄人口达到峰值。“十二五”是我国人力资源最为丰富的时期，劳动年龄人口总量达到峰值，此后缓慢下行，人口抚养比在经历40多年下降后开始上升。
　　老年人口出现第一次增长高峰。20世纪50年代第一次生育高峰出生人口相继进入老年，“十二五”期间我国60岁以上老年人口年均增长800万以上，总量将突破2亿。
　　城镇人口历史性超过农村人口。城镇化率超过50％，城乡人口格局正在发生根本性变化。随着产业转移的加快、中西部城市群的发展，人口流动迁移呈现出新的特点，人口流向趋于多元化。
　　（三）面临的主要问题与矛盾。
　　人口数量对可持续发展的压力仍然较大。我国人口多、底子薄，资源相对不足、环境容量有限，发展不平衡。随着人口继续增长，人口与资源、环境以及经济社会发展的矛盾将进一步显现。劳动年龄人口规模庞大，解决就业问题仍将是长期而艰巨的任务。
　　人口素质正成为提升国家竞争力的瓶颈。国际产业分工的调整、经济发展方式的转变，对人口素质提出了更高的要求，但我国人口素质总体不高，人力资本对经济增长的贡献率远低于发达国家平均水平，正在成为影响我国国际竞争力的重要因素。
　　人口结构性矛盾成为影响经济社会发展的重大问题。我国出生人口性别比长期居高不下，老年人口比重不断提高，人口抚养比开始上升，区域间、城乡间人口发展不平衡，人口结构性矛盾对经济社会发展的影响日益深刻。
　　人口分布不合理影响城乡区域协调发展。人口空间分布与经济布局不协调，与资源环境承载力不适应。城镇化率偏低，流动人口规模庞大，对社会管理和公共服务等带来一系列挑战。
　　家庭结构变化带来新挑战。家庭规模小型化、结构多样化、成员居住分散化趋势明显，导致传统家庭功能弱化，在婚姻、生育、养老等方面出现诸多新问题，人口服务管理相对滞后。
　　总体上看，“十二五”时期是我国人口发展的重大转折期，人口发展的机遇与挑战并存。一方面，经济社会发展仍然面临着人口总量持续增加的压力，人口对经济社会、资源环境的影响更加突出；另一方面，人口各要素关系更趋复杂，素质、结构、分布正在成为影响发展的主要因素。必须从战略上重视人口问题，遵循人口发展规律，充分利用人力资源丰富、社会抚养比低、人口流动活跃的有利时机，全面做好人口工作，为经济社会发展营造良好的人口环境。

　　**二、**总体思路、基本原则和主要目标
　　（一）总体思路。
　　高举中国特色社会主义伟大旗帜，以邓小平理论和“三个代表”重要思想为指导，深入贯彻落实科学发展观，坚定不移走中国特色统筹解决人口问题的道路。按照全面做好人口工作的总体要求，以建设人口均衡型社会为主线，坚持计划生育基本国策，逐步完善政策，稳定低生育水平，提高人口素质，优化人口结构与分布，促进人口长期均衡发展，促进人口与经济社会、资源环境相协调。
　　（二）基本原则。
　　坚持以人为本。把促进人的全面发展作为处理好人口与发展关系的出发点和落脚点。在解决人口问题的过程中，注重保障和改善民生，提高家庭发展能力。
　　坚持统筹协调。强化人口的基础地位，注意把握人口各要素之间、人口与经济社会及资源环境之间的互动关系，构建有利于促进人口长期均衡发展的政策体系。
　　坚持科学指导。把握好人口与经济社会发展的阶段性特点，适时完善人口发展政策，指导地方积极探索统筹解决人口问题的有效方法和途径。
　　坚持创新发展。不断推进人口工作的理论创新、管理创新、服务创新、科技创新，通过体制机制创新，着力解决人口发展中出现的新情况、新问题。
　　（三）主要目标。
　　人口总量目标。低生育水平保持稳定，“十二五”期间，人口年均自然增长率控制在7.2‰以内，全国总人口控制在13.9亿人以内。
　　人口素质目标。教育普及水平进一步提高，15岁以上国民平均受教育年限达到9.3年，新增劳动力平均受教育年限达到13.3年；在职人员培训覆盖面进一步扩大，劳动力素质明显提高，人才队伍进一步壮大。婴儿死亡率降到12‰，孕产妇死亡率降到22／10万，严重多发致残的出生缺陷发生率降低，人均预期寿命达到74.5岁。
　　人口结构目标。出生人口性别结构得到有效改善，全国出生人口性别比下降至115以下。
　　人口分布目标。城镇化率提高约4个百分点，限制开发区域内人口更多地集聚在县城和中心镇，禁止开发区域内人口总量有所减少，人口空间分布趋于合理。流动人口基本公共服务均等化初步实现。
　　民生保障目标。社会就业更加充分，城镇登记失业率控制在5％以内，城乡居民收入大幅提高，家庭发展能力得到增强。覆盖城乡居民的社会保障体系建设加快推进，社会养老服务体系基本建立，养老服务能力明显提升。贫困人口显著减少，残疾人社会保障体系和服务体系框架基本建立，生活状况得到进一步改善。

　　**三、**主要任务
　　（一）坚持计划生育基本国策，稳定低生育水平。
　　全面做好人口和计划生育工作。保持生育政策的连续性、稳定性，更加注重利益引导，更加注重服务关怀，更加注重宣传倡导，全面加强基层基础工作，突出做好重点地区重点人群的人口计生工作。深化人口计生综合改革，着力创新稳定低生育水平、统筹解决人口问题的工作机制和方法，解决影响和制约人口计生工作科学发展的突出问题。坚持依法行政，完善相关法律法规。
　　完善稳定低生育水平相关经济社会政策。将稳定低生育水平的利益导向政策作为保障和改善民生的重要组成部分，纳入政府改善民生行动计划。全面落实法律法规规定的计划生育家庭奖励优惠政策。促进经济社会相关政策与人口计生政策的有机衔接，在就业、社会保障、扶贫开发、征地补偿、集体收益分配等方面，制定对计划生育家庭的倾斜政策。进一步完善以计划生育家庭奖励扶助制度、“少生快富”工程和特别扶助制度为主的优先优惠政策体系，扩大范围、提高扶助标准并建立动态调整机制。
　　完善人口和计划生育公共服务体系。加快人口计生服务体系建设，拓展服务范围。加强人口计生服务基础设施和信息服务配套设施建设。实施人口和计划生育职业体系建设工程，建立职业标准、教育培训、评估认证和职业拓展体系。加强计划生育和生殖健康科技创新基地建设，扶持一批部委级重点实验室。加强新型避孕药具的研究与开发，完善计划生育药具不良反应监测网络。全面提升计划生育优质服务能力，加强生殖健康保健，使人人享有生殖健康服务。
　　（二）完善人口政策，促进人口长期均衡发展。
　　深化人口长期均衡发展战略研究。按照科学发展观以及加快经济发展方式转变的要求，充分考虑人口变化对经济社会发展的长期性、基础性影响，准确把握人口变动趋势，深入研究不同阶段的人口政策取向及主要任务，为人口决策提供支撑。
　　坚持和完善现行生育政策。进一步完善宣传教育、依法管理、村（居）民自治、优质服务、政策推动、综合治理的长效工作机制，确保低生育水平的稳定。着眼长远发展，逐步完善政策，确保人口中长期战略目标的实现。
　　形成人口政策合力。进一步完善人口计生、人力资源开发、男女平等、人口老龄化以及人口迁移流动等方面政策，制定促进家庭发展、主体功能区建设等方面的人口配套政策，加快形成统筹解决人口问题的政策体系。
　　（三）着力提高人口素质，加快人口大国向人力资源强国转变。
　　提高出生人口素质。有关部门按照职责分工，加强协作，积极落实出生缺陷三级预防措施，加大出生缺陷干预力度。开展易为广大群众接受的宣传和引导活动，加强婚前、孕前咨询指导。组织实施计划生育生殖健康促进工程，做好健康教育、优生咨询、高危人群指导、孕前筛查、营养素补充等优生服务工作。逐步扩大国家免费孕前优生健康检查项目试点范围，探索建立国家免费孕前优生健康检查制度。建设全国产前诊断网络，推进新生儿疾病筛查、诊断和治疗工作。开展出生缺陷发生机理和防治技术研究，加强出生缺陷防治重点实验室建设。提高助产服务能力，进一步降低孕产妇和婴儿死亡率。
　　提高人口健康素质。普及健康教育，积极倡导健康文明的生活方式，大力推进全民健身运动。全面加强公共卫生服务体系建设，健全医疗保障和服务体系，完善基本医疗制度。充分利用基层医疗卫生和计划生育服务网络，形成以预防为主、防治结合的公共卫生服务体系，逐步缩小城乡居民基本公共卫生服务差距。继续加强性病、艾滋病的防治工作，全面实施慢性病综合防控，最大限度地控制和减少传染病、地方病的发生和传播。加强心理卫生和精神健康工作。
　　提升国民教育水平。贯彻落实《国家中长期教育改革和发展规划纲要（2010-2020年）》，深化教育体制改革，全面实施素质教育，大力促进教育公平，加快构建覆盖城乡的基本公共教育服务体系。巩固提高义务教育普及成果，全面提高质量和水平，促进义务教育均衡发展。加强婴幼儿早期教育。积极发展学前教育，构建“广覆盖、保基本、多形式”的学前教育体系。大力发展职业教育，巩固规模，提高质量，建立支撑现代产业发展的职业教育体系。全面提高高等教育质量，实现从以规模扩张为基本特征的外延式发展向以提高质量为核心的内涵式发展转变，办出具有中国特色、世界水平的现代高等教育。加快发展多样化的继续教育，重视和支持特殊教育，继续发展老年教育，推动民族教育加快发展。健全国家资助政策体系，扶助家庭经济困难学生完成学业。加强青少年健康人格教育、独生子女社会行为教育。加强道德素质和诚信教育，提高国民素养。
　　加强人才队伍建设。加大专业技术人才和高技能人才培养力度。优化人才培养结构，突出培养造就创新型科技人才，重视开发领军人才，大力开发经济社会发展急需紧缺专门人才，提高应用型、复合型、技能型人才培养规模和水平。建立和完善职业资格证书制度，对城乡有就业要求和培训愿望的劳动者参加职业技能培训给予培训费补贴，推进中等职业教育免费进程。加强农村实用人才的培养，提高农民工的就业能力。营造人才脱颖而出的环境，实施更加积极的就业政策，充分发挥劳动力资源丰富的优势，加快人口大国向人力资源强国转变，为经济社会发展提供持久动力。
　　（四）引导人口有序流动，促进人口合理布局。
　　优化人口布局。实施与主体功能区相配套的人口政策，促进形成合理的人口分布格局。积极推进资源环境承载能力较强、经济发达的城市化地区吸纳和集聚人口，引导人口超载的重要生态地区人口自愿、平稳、有序转移。深入开展人口空间分布研究，做好人口发展规划和主体功能区规划的衔接协调。研究促进人口有序流动、合理分布的政策，探索优化人口分布的有效途径。
　　积极稳妥推进城镇化。将符合条件的农业转移人口逐步转为城镇居民作为推进城镇化的重要任务。积极稳妥推进户籍管理制度改革，特大城市要合理控制人口规模，大中城市要加强和改进人口管理，继续发挥吸纳人口的重要作用，中小城市和小城镇要根据实际放宽落户条件。坚持因地制宜、分步推进，按规定把有合法稳定职业并有合法稳定住所（含租赁）的农村人口逐步转为城镇居民。构建城镇化战略格局，完善城镇基础设施，增强城镇承载能力，改善人居环境。统筹考虑人口变化和城镇化趋势，优化城乡公共服务设施布局。加快推进社会主义新农村建设，促进城乡经济社会发展一体化。
　　创新流动人口服务管理体制机制。建立全国流动人口生存发展状况、分布的动态监测体系。在国家综合配套改革试验区进行改革试点，积极探索符合时代要求的人口服务管理体制。逐步实行暂住人口居住证制度。在农民工流动较为集中的地区建立农民工综合服务中心，为流动人口提供“一站式”服务。建立农民工基本培训补贴制度，增加对农民工的技能培训和就业服务。将与企业建立稳定劳动关系的农民工纳入城镇职工基本养老和医疗保险。多渠道多形式改善农民工居住条件，鼓励采取多种方式将符合条件的农民工纳入城镇住房保障体系。坚持以输入地政府管理为主，以全日制公办中小学为主，确保进城务工人员随迁子女平等接受义务教育。研究制定进城务工人员随迁子女接受义务教育后在当地参加升学考试的办法。巩固完善流动人口计划生育“一盘棋”工作机制，建立流动人口计划生育基本公共服务均等化的保障机制。
　　（五）健全养老保障和服务体系，积极应对人口老龄化。
　　探索具有中国特色的应对人口老龄化新路子。深化应对人口老龄化战略研究，实施积极应对人口老龄化战略，制定与其他经济社会政策互相衔接、相互支撑的应对人口老龄化政策体系，推进老年人权益保障法制化进程。提高老年人口的素质和技能，充分开发老年人力资源，鼓励老年人参与经济社会活动。加强组织领导，健全党政主导、老龄委协调、部门尽责、社会参与、全民关怀的大老龄工作格局。
　　完善养老保障制度。以基本养老保险为重点，加快完善城乡居民社会养老保障制度。实现新型农村社会养老保险制度和城镇居民社会养老保险制度全覆盖，完善城镇职工基本养老保险制度，全面落实城镇职工基本养老保险省级统筹，实现基础养老金全国统筹，切实做好城镇职工基本养老保险关系接续工作。城镇参加基本养老保险人数达到3.57亿人，60岁以上非就业城镇居民全部享受基础养老金待遇。鼓励有条件的地区在养老保险基础上，进一步加强养老保障工作，积极探索为独生子女父母、无子女和失能老人提供必要的养老服务补贴和老年护理补贴。
　　加快养老服务体系建设。注重发挥家庭和社区功能，建立以居家为基础、社区为依托、机构为支撑的社会养老服务体系，每千名老人拥有养老床位达到30张。加强养老服务标准、行业规范和管理制度建设，建立养老机构准入、退出与监管机制。优先发展社会养老服务，加强老年人宜居环境建设，增加社区老年活动场所和便利设施。
　　（六）综合治理出生人口性别比偏高问题，促进社会性别平等。
　　坚持男女平等基本国策。提高社会性别平等意识，清理涉及社会性别歧视的法规政策。指导村（居）民自治组织修订完善自治章程或村规民约，在扶贫济困、慈善救助、贴息贷款、就业服务、项目扶持、村集体收益分配等方面对计划生育家庭女儿户予以倾斜。深入推进关爱女孩行动，进一步开展“幸福工程”和“春蕾计划”等社会公益性活动，充分发挥社会组织在贫困母亲救助和女童健康成长帮助中的重要作用。
　　推动妇女儿童事业全面发展。贯彻《[国务院关于印发中国妇女发展纲要和中国儿童发展纲要的通知](https://www.pkulaw.com/chl/0fe9880c331fcc87bdfb.html)》（国发〔2011〕24号），切实保障妇女合法权益，加强未成年人保护。促进妇女就业创业，提高妇女参与经济发展和社会管理能力。严厉打击暴力侵害妇女、拐卖妇女儿童、弃婴等违法犯罪行为。切实解决流动儿童、留守儿童、孤残儿童和流浪未成年人救助等问题。
　　加强出生人口性别比综合治理。建立健全国家和省级部门间协调机制，把促进社会性别平等、综合治理出生人口性别比偏高问题列入地方各级人口和计划生育领导小组成员单位的职责范围。规范人工终止妊娠药品和计划生育手术器械经营管理，完善医学需要的胎儿性别鉴定和人工终止妊娠登记、孕产期全程服务管理制度。严厉打击非医学需要的胎儿性别鉴定和选择性别人工终止妊娠行为。完善出生统计监测体系，全面实施出生实名登记制度，建立部门间出生人口信息收集和共享机制。强化区域协作和重点区域的治理，在出生人口性别比严重偏高的连片地区，建立区域协查制度。在重点区域探索建立跨省区出生人口信息共享制度和出生性别监测预警机制。
　　（七）提高家庭发展能力，促进家庭和谐幸福。
　　建立健全家庭发展政策。稳定家庭功能，在优生优育、家庭教育、子女成才、抵御风险、生殖健康、家庭致富以及养老保障等方面，加快建立和完善提高家庭发展能力的政策体系。加大对孤儿监护人家庭、老年人家庭、残疾人家庭、留守家庭、流动家庭、受灾家庭和其他特殊困难家庭的扶持力度。进一步完善工伤、生育等保险制度，合理提高低保标准和补助水平，加强城乡社会救助体系建设，积极发展社会福利和慈善事业。提高住房保障水平。完善生育关怀制度，将计划生育节育手术并发症三级以上人员纳入计划生育家庭特别扶助制度范围。免费为符合条件的育龄妇女提供再生育技术服务。研究出台有利于促进计划生育家庭成员就业、创业、勤劳致富的扶持政策。
　　着力提高家庭服务能力。大力发展家庭服务业，逐步建立比较健全的惠及城乡居民的家庭服务体系。在全员人口信息系统中，加强家庭信息采集和管理，为家庭发展政策的制定和实施提供依据。探索建立以家庭为中心的人口计生公共服务体系，开展婚育指导、家庭初级保健、儿童早期发展、家庭教育指导，以及对计划生育家庭、空巢家庭、流动家庭、留守家庭的关怀服务等。
　　大力推进新型家庭人口文化建设。以婚育新风进万家活动、关爱女孩行动、新农村新家庭计划、幸福工程为载体，广泛宣传人口计生政策。开展幸福家庭创建活动。倡导婚姻自由平等、生殖健康、优生优育、社会性别平等的观念，倡导积极健康、负责任的婚育行为，倡导低消耗、低污染的家庭生活方式。大力弘扬尊老爱幼、邻里互助的社会风尚。加强流动人口聚集区多元文化交流，促进流动人口融入当地社会。加强农村人口文化设施建设，纳入新农村建设整体规划。加强少数民族人口文化建设。
　　（八）健全残疾人保障和服务体系，支持残疾人事业发展。
　　健全残疾人社会保障体系。鼓励残疾人普遍加入基本医疗保险和基本养老保险，逐步将残疾人基本康复医疗按规定纳入基本医疗保障范围。将符合条件的残疾人家庭优先纳入基本住房保障制度，继续实施“阳光安居”农村贫困残疾人家庭危房改造工程。逐步建立贫困残疾人生活补助和重度残疾人护理补贴制度。支持残疾人辅助器具的研发、生产和适配服务，制定政府补贴的残疾人基本型辅助器具目录。
　　完善残疾人服务体系。实施重点康复工程，为贫困残疾人提供康复服务。全面开展社区康复工作，推进残疾人“人人享有康复服务”。制定实施国家残疾预防行动计划。继续实施“阳光家园计划”。健全残疾人托养服务体系，大力发展居家助残服务。积极开展无障碍建设。实施残疾人文化建设和自强健身工程，丰富残疾人精神文化生活。
　　促进残疾人就学就业。改善特殊教育办学条件，逐步实行残疾学生高中阶段免费教育，加大对家庭困难残疾学生的资助力度。为残疾人免费提供公共就业服务，对残疾人参加职业技能培训和职业技能鉴定的，按规定给予补贴。完善残疾人就业促进和保护政策措施，加强残疾人就业援助，积极开发适合残疾人的公共就业岗位。加强对农村残疾人的生活救助和生产扶助。

　　**四、**主要保障措施
　　（一）加强组织领导和统筹协调。各地区、各有关部门要把统筹解决人口问题、建设人口均衡型社会列入重要议事日程，把人口计生工作列入经济社会发展总体规划，纳入改善民生的总体部署。构建党委领导、政府负责、社会协同、公众参与的人口计生工作格局，健全领导机制、协调机制、监督机制和问责机制，坚持人口计生工作党政一把手亲自抓、负总责，完善目标管理责任制和“一票否决”制度。坚持人口发展规划先行，其他专项规划制定应充分考虑人口因素影响；重大经济社会政策出台前，应开展对人口发展影响的评估。
　　（二）健全人口监测体系。建设国家人口基础信息库，实施“全员人口统筹管理信息系统”工程，建立人口信息动态采集和更新机制，科学监测和评估人口发展状况。构建科学的人口发展指标体系，规范人口统计口径。建立完善部门间人口信息共享制度和人口统计信息沟通机制，及时发布人口总量、结构、分布预测、预警信息，为科学决策、人口综合服务管理提供信息支撑。
　　（三）完善人口服务管理体系。完善人口计生服务管理体系，优化公共资源配置，以全员人口信息为基础，构建人口管理和公共服务平台。充分发挥人口和计划生育公共服务网络的作用，探索建立社区人口和家庭服务中心。加强人口计生队伍建设，完善职业体系，造就一支高素质的人口管理和服务人才队伍。
　　（四）建立健全投入保障机制。建立“财政为主、稳定增长、分类保障、分级负担、城乡统筹”的人口和计划生育投入保障机制，确保人口和计划生育财政投入增长幅度高于经常性财政收入增长幅度；区别不同重大项目，分别确定中央和地方资金的分担办法；按照“费随人走”的原则，以常住人口为依据进行中央财政对地方的转移支付；完善绩效挂钩、以奖代投（补）激励机制。鼓励民间资本投入人口发展领域。进一步加大对革命老区、民族地区、边疆地区、贫困地区人口发展的政策支持和资金扶持力度。
　　（五）加大宣传与国际交流力度。深入开展人口国情、人口政策和人口发展规划的宣传，充分发挥新闻媒体作用，正确引导舆论，在全社会营造有利于人口计生事业发展的氛围。发挥中国计划生育协会等社会团体作用，动员社会力量共同做好人口计生工作。建立和完善国际交流与合作机制，积极参与、引导有关国际规则的制定。
　　（六）加强规划实施的监测评估。地方各级人民政府、各有关部门共同做好人口发展规划的实施工作，制定本地区、本部门的具体实施方案和年度计划。加强对规划实施情况的动态监测与跟踪分析，及时发现和解决规划执行过程中存在的问题，定期组织对规划实施情况的检查，开展规划执行情况期中和期末评估，确保规划各项任务落到实处。

© 北大法宝：（[www.pkulaw.com](https://www.pkulaw.com/)) 专业提供法律信息、法学知识和法律软件领域各类解决方案。北大法宝为您提供丰富的参考资料，正式引用法规条文时请与标准文本核对。
欢迎查看所有[产品和服务](https://www.pkulaw.com/helps/2.html)。法宝快讯：[如何快速找到您需要的检索结果？](https://www.pkulaw.com/helps/70.html)[法宝](https://www.pkulaw.com/helps/69.html)V6有何新特色？

扫描二维码阅读原文

**原文链接：**<https://www.pkulaw.com/chl/27871550f2830f80bdfb.html>
